# Disseminated Native Tricuspid Valve Infective Endocarditis and Vertebral Osteomyelitis Secondary to Veillonella dispar in a Patient Who Injects Drugs

**DOI:** 10.7759/cureus.17989

**Published:** 2021-09-15

**Authors:** Love Shah, Stephen Pylypchuk, Shaqil Peermohamed

**Affiliations:** 1 Internal Medicine, University of Saskatchewan College of Medicine, Saskatoon, CAN; 2 Internal Medicine/Cardiology, University of Saskatchewan College of Medicine, Saskatoon, CAN; 3 Internal Medicine/Infectious Disease, University of Saskatchewan College of Medicine, Saskatoon, CAN

**Keywords:** tricuspid valve endocarditis, veillonella, vertebral osteomyelitis, injection drug use, infective endocarditis

## Abstract

We present the case of a 50-year-old man presenting with fever, back pain, persistent bacteremia with *Veillonella dispar*, echocardiographic evidence of a tricuspid valve vegetation increasing in size, and magnetic resonance imaging suggesting new vertebral osteomyelitis. He was successfully treated with intravenous ceftriaxone for six weeks. Deep-seated infections secondary to *Veillonella* species are rare, but cases of endocarditis, osteomyelitis, and meningitis have been reported in the literature. Given *Veillonella* species are normal human commensals present in the oropharyngeal flora, we suspect our patient developed native tricuspid valve endocarditis and vertebral osteomyelitis as a complication of either poor dentition or contaminated injection drug use paraphernalia and subsequent hematogenous seeding.

## Introduction

*Veillonella* species are known to be slow-growing, anaerobic, Gram-negative cocci that are normal human commensals present in the oropharyngeal, gastrointestinal, and genitourinary regions [1,2]. The genus *Veillonella* has approximately 13 species, of which six have been isolated from the oral mucosa and predominate in patients with poor oral hygiene [3]. *Veillonella* is typically a benign organism; however, it has been reported in association with deep-seated infections, including endocarditis, osteomyelitis, and meningitis [2]. Due to the rare pathogenicity of *Veillonella* species, its antibiotic susceptibility patterns are not well studied; however, it is generally accepted to be susceptible to penicillins and cephalosporins [4]. Here, we describe the first case of a patient who injects drugs with infective endocarditis and vertebral osteomyelitis secondary to *Veillonella dispar*.

## Case presentation

A 50-year-old man presented to the emergency room with several months of intermittent fever and progressive lower back pain. He described a mechanical fall one week prior to this admission. He had a medical history of alcohol abuse, intravenous drug use, hepatitis C infection, and native tricuspid valve infective endocarditis secondary to methicillin-resistant *Staphylococcus aureus* (MRSA) five months prior to his current hospital admission. At that time, his transthoracic echocardiogram showed a tricuspid valve vegetation measuring 11 × 6 mm with severe tricuspid regurgitation. He was partially treated with intravenous vancomycin followed by daptomycin with serial negative blood cultures. Magnetic resonance imaging (MRI) of his spine at that time showed no evidence of vertebral osteomyelitis or epidural abscess.

**Figure 1:**
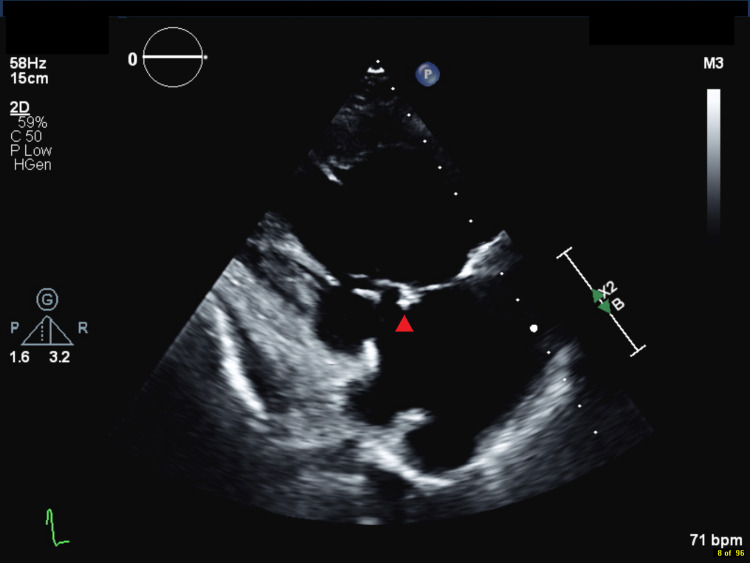
Right ventricular inflow imaging of the tricuspid valve with evidence of an independently mobile mass/vegetation (red arrowhead) on the atrial aspect of the tricuspid valve, which appears smaller in size compared to the previous echocardiogram, now measuring 7 × 6 mm.

Two months prior to the current hospital admission, he presented to the emergency room with back pain. At this time, his blood cultures were positive for the growth of *V. dispar*, which was identified by matrix-assisted laser desorption/ionization-time of flight mass spectrometry. A transthoracic echocardiogram was performed prior to him leaving the hospital against medical advice and showed that the tricuspid valve vegetation was now smaller in size, measuring 7 × 6 mm (Figure 1). He received no antimicrobial treatment at this time.

During his current hospital admission, he was visibly agitated on physical examination and described significant lower back pain. His initial vital signs included a temperature of 39.5 degrees Celsius, heart rate of 120 beats per minute, respiratory rate of 20 breaths per minute, oxygen saturation of 97% on ambient room air, and blood pressure of 106/68 mmHg. His neck was supple and his neurological examination demonstrated normal tone throughout his upper and lower extremities. Strength in his right lower extremity was 3/5 for ankle plantar flexion and dorsiflexion, 2/5 for knee extension and flexion, and 3/5 for hip extension and flexion. His strength was 4/5 throughout his left lower extremity. His sensation was normal apart from decreased light touch sensation to his medial right gastrocnemius region. He had spinal and paraspinal tenderness in his mid and lower back. His reflexes were 2+ in his knees and plantars were down-going bilaterally. He had a grade III/VI systolic murmur best heard at the left lower sternal border. Air entry was equal and clear bilaterally with fine crackles to his right lower lung zone. No other peripheral stigmata of infective endocarditis were present on examination. Examination of the oral cavity showed he was missing upper teeth with overall poor dentition. Initial laboratory investigations are summarized in Table 1.

**Table 1:** Summary of initial laboratory investigations.

Laboratory investigation	Patient result	Normal range
Leukocyte count	17.9 × 10^9^/L	4.0–11.0 × 10^9^/L
Neutrophil count	16.9 × 10^9^/L	1.5–7.5 × 10^9^/L
Hemoglobin	92 g/L	135–180 g/L
Platelet count	208 × 10^9^/L	150–400 × 10^9^/L
Creatinine	189 μmol/L	60–104 μmol/L
C-reactive protein	67.3 mg/L	0.0–7.0 mg/L

**Figure 2:**
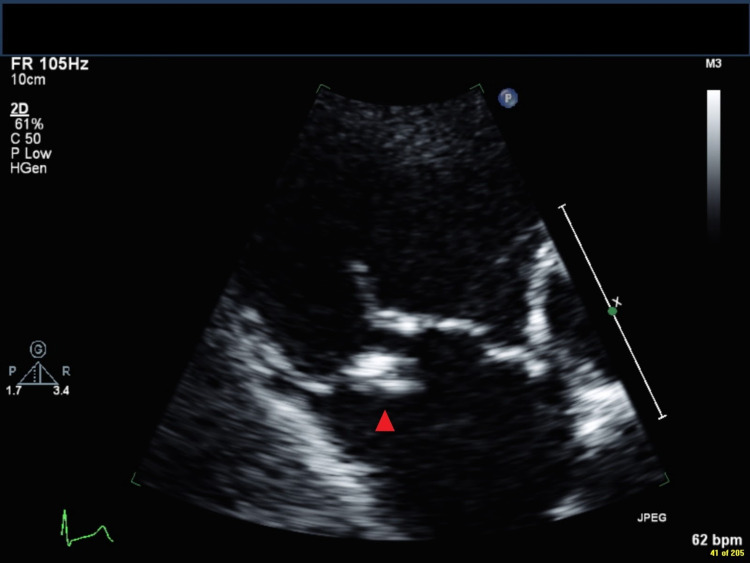
Transthoracic echocardiogram in parasternal right ventricular inflow view showing the vegetation (red arrowhead), which has increased in size to 11 × 6 mm, adherent to the atrial aspect of the tricuspid septal leaflet.

**Figure 3:**
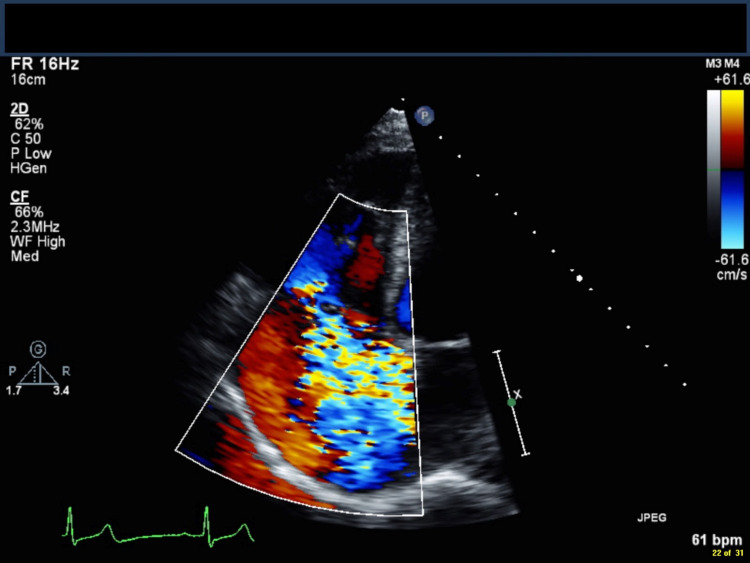
A transthoracic echocardiogram in apical four-chamber view showing severe, torrential central tricuspid regurgitation (blue jet).

His electrocardiogram was normal. During his hospital admission, two sets of blood cultures obtained 96 hours apart were again positive for the growth of *V. dispar*. Given his persistent bacteremia, a transthoracic echocardiogram was performed which showed growth in the size of the vegetation previously noted on his tricuspid valve, now measuring 11 × 7 mm. There were also signs of torrential tricuspid regurgitation including a vena contracta width greater than 0.7 cm, a central jet that was filling greater than 50% of the right atrium, and a triangular-shaped continuous-wave Doppler signal which was as dense as the inflow signal along with evidence of systolic hepatic vein flow reversal (Figures 2, 3).

**Figure 4:**
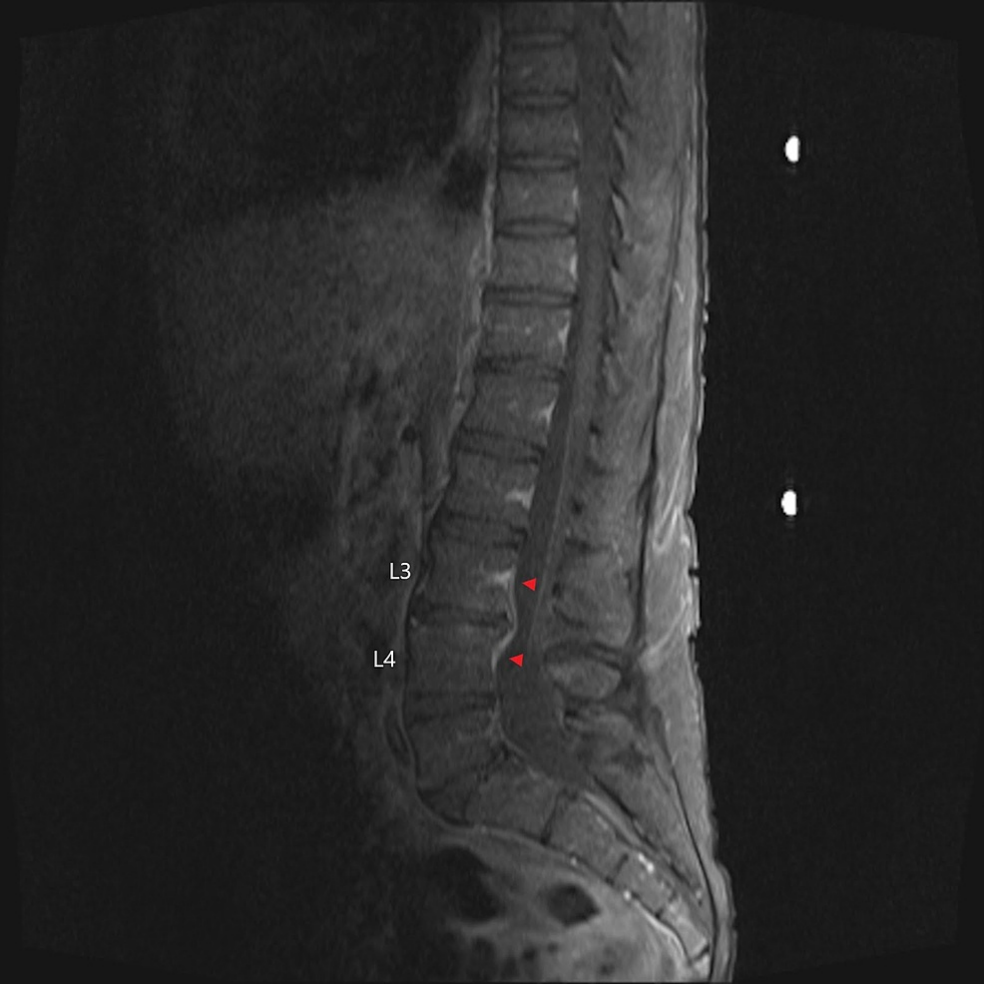
Pre-treatment T1 fat-suppressed post-gadolinium MRI sagittal view showing signal enhancement of the L3/L4 vertebral bodies (red arrowheads). MRI: magnetic resonance imaging

An MRI scan of the spine showed early discitis/osteomyelitis of L3-L4 with a right paravertebral and right paracentral anterior epidural phlegmon with no abscess (Figure 4). The neurosurgical team assessed the patient and recommended conservative management with ongoing antimicrobial therapy and physiotherapy, and monitoring for any worsening neurological deficits. In addition, given his poor dentition, a computed tomography (CT) scan of the brain, head, and neck was arranged, which showed no obvious evidence of an abscess collection within the neck, oropharynx, or brain. Furthermore, no septic pulmonary emboli were seen on imaging.

Susceptibility testing was performed on his *V. dispar* isolate using broth microdilution and showed susceptibility to penicillin (minimum inhibitory concentration [MIC] < 0.5), amoxicillin-clavulanate (MIC < 4/2), and metronidazole (MIC < 8) compared against breakpoints according to the Clinical and Laboratory Standards Institute. He was treated with intravenous ceftriaxone for six weeks with clinical improvement noted within a few days following his admission. The patient reported near-complete resolution of his back pain and his repeat C-reactive protein level toward the end of therapy was 3.8 mg/L (normal 0.0-7.0 mg/L). Subsequently, he developed a central-line-associated bloodstream infection secondary to *Candida dubliniensis* with no evidence of endophthalmitis, for which he received a two-week course of intravenous caspofungin.

**Figure 5:**
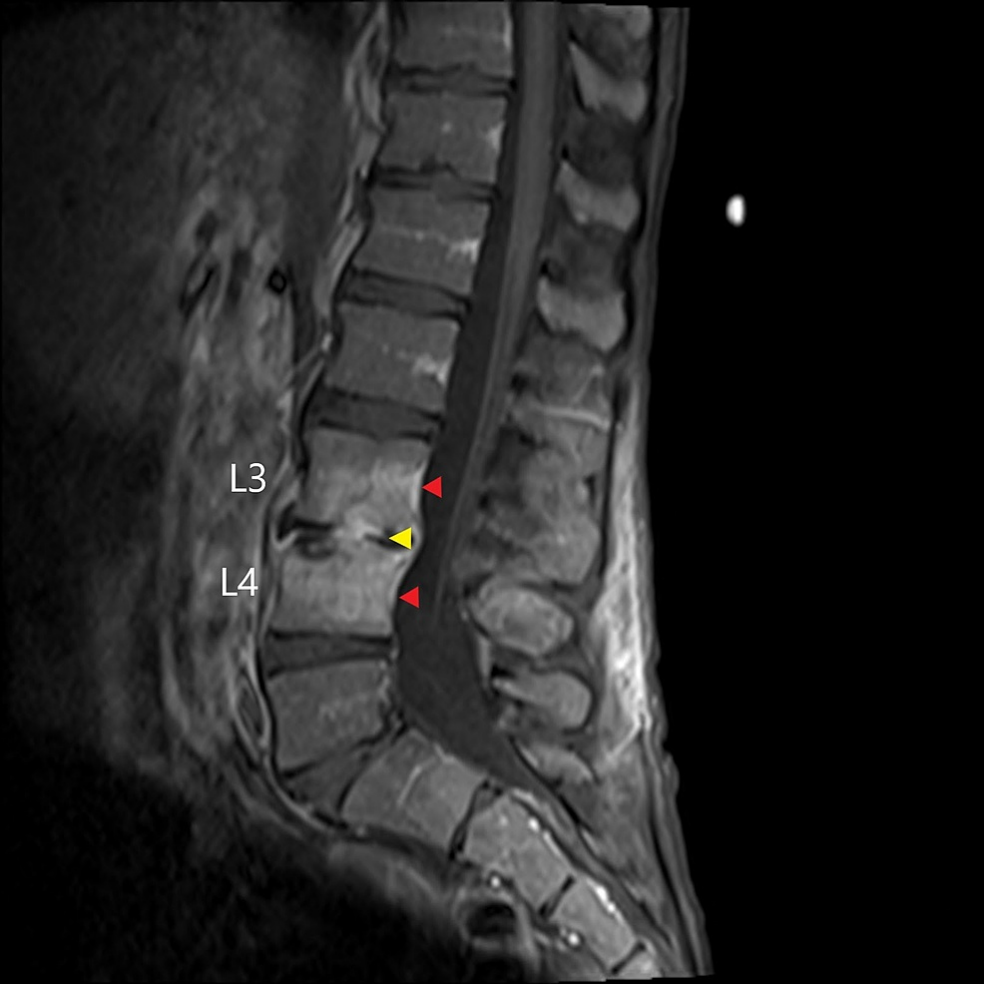
Post-treatment T1 fat-suppressed post-gadolinium MRI sagittal view demonstrating enhancement of the L3/L4 disc space (yellow arrowhead) and enhancement of the adjacent L3 and L4 vertebral bodies (red arrowheads). MRI: magnetic resonance imaging

A repeat MRI was arranged after his therapy which showed resolution of the anterior epidural phlegmon but interval worsening of the L3-L4 discitis/osteomyelitis with a new enhancement of the L3 and L4 facets without effusion (Figure 5). A subsequent CT-guided lumbar spine biopsy of the L3/L4 region was performed, and bacterial, fungal, and mycobacterial cultures were negative. A repeat transthoracic echocardiogram was planned to establish a new baseline for his valvular anatomy given his risk of recurrent endocarditis; however, the patient left against medical advice.

## Discussion

*Veillonella* species are small, Gram-negative cocci that grow in obligate anaerobic environments. The genus was first isolated in 1898 by Veillon and Zuber from an appendiceal abscess [1]. The most common *Veillonella* species isolated is *V. parvula*, which was originally named *Staphylococcus parvulus* [4,5]. *Veillonella* species are normal human flora found predominantly in the oropharyngeal, gastrointestinal, and genitourinary tracts [2].

*Veillonella *species can adhere to other oral bacterial flora and can subsequently create a polymicrobial bacterial network of multiple species [6]. This relationship which is known as intergeneric coaggregation is important in forming econiches that colonize various oral surfaces [7]. There has been increased interest in the pathogenicity of *Veillonella* species and their association with deep-seated infections [8]. The most common underlying disease and risk factors associated with deep-seated infections secondary to *Veillonella* species include immunodeficiencies, drug abuse, and malignancy [8]. *Veillonella* species have been previously described in association with deep-seated infections such as meningitis, endocarditis, and osteomyelitis secondary to hematogenous seeding [8-15]. *Veillonella* species such as *V. parvula* have been described in association with vertebral osteomyelitis and diabetic foot osteomyelitis, often with concurrent bacteremia [4].

Cases of both native and prosthetic valve endocarditis have been reported due to *Veillonella* species, including *V. alcalescens*, *V. dispar*, and *V. parvula*. In one case series of patients with infective endocarditis secondary to *Veillonella* species, the authors describe nine patients with endocarditis involving the mitral, aortic, or tricuspid valves [2]. In this case series, four patients had native valve endocarditis, four had prosthetic valve endocarditis, and one was unspecified [2]. Patients who inject drugs are at an increased risk of polymicrobial endocarditis, with one prior case report describing a patient who injected drugs and was found to have tricuspid valve endocarditis and bacteremia with *Veillonella* species, *Actinomyces odontolytica*,and *Prevotella melaninogenica*. The authors described that this patient most likely acquired these organisms due to a habit of licking needles before injection [15]. Given *Veillonella *species are often isolated in polymicrobial infections, it is challenging to determine their pathogenic role and mortality rates associated with bacteremia and deep-seated infections. Furthermore, the rarity of case reports describing monomicrobial, deep-seated infections due to *Veillonella* species makes it challenging to determine both the incidence and optimal antimicrobial treatment [2,4].

While the optimal antimicrobial regimen and duration of treatment for endocarditis caused by *Veillonella* species is unclear, prior cases have described successful treatment with approximately six weeks of a variety of antimicrobials, including penicillin, ampicillin, first and third-generation cephalosporins, metronidazole, clindamycin, or aminoglycosides, used in monotherapy or combination therapy [2]. Surgical intervention may also be required, especially in the setting of prosthetic valve endocarditis [2]. Previously reported cases of vertebral and non-vertebral osteomyelitis secondary to *Veillonella* species also describe various antimicrobial treatment strategies with the duration of treatment ranging from four to eleven weeks [4].

In our patient, although the source of his bacteremia with *V. dispar* is unclear, we speculate it could have been related to salivary contact with his injection paraphernalia before injection use. We suspect that our patient’s percutaneous vertebral biopsy was negative for bacterial growth given he had received six weeks of ceftriaxone. However, his bacteremia with *V. dispar* coinciding with his back pain supports this being the causative pathogen of his L3-L4 vertebral osteomyelitis.

## Conclusions

Deep-seated infections such as endocarditis and osteomyelitis secondary to *Veillonella* species are infrequently reported but have been described in patients with poor oral hygiene, periodontal disease, and autoimmune disease. Patients who inject drugs are potentially at an increased risk of deep-seated infections secondary to *Veillonella* species through multiple mechanisms, including hematogenous seeding secondary to injection drug use with needle-saliva contact from needle-licking or salivary solvent use, or hematogenous seeding due to poor underlying dentition. Our case illustrates how patients who inject drugs are at an increased risk of recurrent endocarditis and the importance of recognizing *Veillonella* species as a rare but serious cause of endocarditis and osteomyelitis in patients who inject drugs.
